# Comparative Study of Free-Roaming Domestic Dog Management and Roaming Behavior Across Four Countries: Chad, Guatemala, Indonesia, and Uganda

**DOI:** 10.3389/fvets.2021.617900

**Published:** 2021-03-04

**Authors:** Charlotte Warembourg, Ewaldus Wera, Terence Odoch, Petrus Malo Bulu, Monica Berger-González, Danilo Alvarez, Mahamat Fayiz Abakar, Filipe Maximiano Sousa, Laura Cunha Silva, Grace Alobo, Valentin Dingamnayal Bal, Alexis Leonel López Hernandez, Enos Madaye, Maria Satri Meo, Abakar Naminou, Pablo Roquel, Sonja Hartnack, Salome Dürr

**Affiliations:** ^1^Veterinary Public Health Institute, Vetsuisse Faculty, University of Bern, Bern, Switzerland; ^2^Graduate School for Health Sciences, University of Bern, Bern, Switzerland; ^3^Kupang State Agricultural Polytechnic (Politeknik Pertanian Negeri Kupang), West Timor, Indonesia; ^4^College of Veterinary Medicine, Animal Resources and Biosecurity, Makerere University, Kampala, Uganda; ^5^Center for Health Studies, Universidad del Valle de Guatemala, Guatemala City, Guatemala; ^6^Swiss Tropical and Public Health Institute, Basel, Switzerland; ^7^Institut de Recherche en Elevage pour le Développement, N'Djamena, Chad; ^8^Faculty of Veterinary Medicine, University of Lisbon, Lisbon, Portugal; ^9^Animal Health Division, Agricultural Department of Sikka Regency, Flores, Indonesia; ^10^Section of Epidemiology, Vetsuisse Faculty, University of Zurich, Zurich, Switzerland

**Keywords:** free-ranging dog, home range, predictor, global positioning system, dog collar

## Abstract

Dogs play a major role in public health because of potential transmission of zoonotic diseases, such as rabies. Dog roaming behavior has been studied worldwide, including countries in Asia, Latin America, and Oceania, while studies on dog roaming behavior are lacking in Africa. Many of those studies investigated potential drivers for roaming, which could be used to refine disease control measures. However, it appears that results are often contradictory between countries, which could be caused by differences in study design or the influence of context-specific factors. Comparative studies on dog roaming behavior are needed to better understand domestic dog roaming behavior and address these discrepancies. The aim of this study was to investigate dog demography, management, and roaming behavior across four countries: Chad, Guatemala, Indonesia, and Uganda. We equipped 773 dogs with georeferenced contact sensors (106 in Chad, 303 in Guatemala, 217 in Indonesia, and 149 in Uganda) and interviewed the owners to collect information about the dog [e.g., sex, age, body condition score (BCS)] and its management (e.g., role of the dog, origin of the dog, owner-mediated transportation, confinement, vaccination, and feeding practices). Dog home range was computed using the biased random bridge method, and the core and extended home range sizes were considered. Using an AIC-based approach to select variables, country-specific linear models were developed to identify potential predictors for roaming. We highlighted similarities and differences in term of demography, dog management, and roaming behavior between countries. The median of the core home range size was 0.30 ha (95% range: 0.17–0.92 ha) in Chad, 0.33 ha (0.17–1.1 ha) in Guatemala, 0.30 ha (0.20–0.61 ha) in Indonesia, and 0.25 ha (0.15–0.72 ha) in Uganda. The median of the extended home range size was 7.7 ha (95% range: 1.1–103 ha) in Chad, 5.7 ha (1.5–27.5 ha) in Guatemala, 5.6 ha (1.6–26.5 ha) in Indonesia, and 5.7 ha (1.3–19.1 ha) in Uganda. Factors having a significant impact on the home range size in some of the countries included being male dog (positively), being younger than one year (negatively), being older than 6 years (negatively), having a low or a high BCS (negatively), being a hunting dog (positively), being a shepherd dog (positively), and time when the dog was not supervised or restricted (positively). However, the same outcome could have an impact in a country and no impact in another. We suggest that dog roaming behavior is complex and is closely related to the owner's socioeconomic context and transportation habits and the local environment. Free-roaming domestic dogs are not completely under human control but, contrary to wildlife, they strongly depend upon humans. This particular dog–human bound has to be better understood to explain their behavior and deal with free-roaming domestic dogs related issues.

## Introduction

Free-roaming domestic dog (FRDD) movements have been studied in various countries across the world. Previous studies involved dogs living in Australia (1–8); Latin America, including Brazil (9, 10), Mexico (11, 12), and Chile (13–16); and Asia, including India (17), Tibet (18), and Kyrgyzstan (19). To the best of our knowledge, no study investigated FRDD roaming behavior in Africa so far. Often, data were collected using Global Positioning System (GPS) tracking devices (1–4, 6–8, 11–16, 18–20), but other tools, such as capture–recapture (10, 21) or interviews (9), were also used to collect data on FRDD roaming behavior. Home range (2, 14, 17, 19), the area a dog commonly uses for normal activities, such as breeding or foraging, and distance from home (12, 13, 20) were applied to describe and investigate dog roaming behavior. These studies improved knowledge on FRDD behavior by investigating dog movements in relation to their habitat (15, 16), interactions with wildlife (7, 12), impact of dog characteristics (6, 8, 10, 13, 22), or sterilization (10, 14) on dog roaming behavior.

Studies investigating predictors for roaming are of particular interest, since they could be used to inform infectious disease control measures. For example, during vaccination campaigns, additional effort could be put toward dogs having larger home ranges or roaming further away from home since they might be in contact with a higher number of dogs or could spread infectious disease over longer distances (23). Once characteristics of those dogs are identified, their owners could be targeted by awareness raising campaigns addressing vaccination benefits. Rabies, a neurological disease caused by the Rabies Virus (RABV), almost always fatal after the onset of the symptoms, is the disease the most commonly investigated in studies on FRDD. FRDD play an important role in rabies spread since dogs are considered as the main source for rabies transmission to humans (24). However, dog behavior has also been investigated in regard to other dog-transmitted zoonotic diseases, such as echinococcosis (18, 19), Leishmaniasis (25, 26), or Rocky Mountain spotted fever (11), because of their impact on human health. The objectives of those studies include the refinement of current control strategies in endemic areas, preventing disease incursion in countries free from specific diseases (e.g., rabies in Australia) and informing dog population management programs (20, 23, 27).

Previous studies on predictors are generally restricted to a specific geographic area, and findings between the studies can be contradictory. For example, in some studies, sex was identified as a predictor for roaming (6, 8, 10, 18, 22) while other studies did not detect any difference based on sex (14, 19, 20). Among the studies that identified sex as a predictor, some concluded that male dogs were roaming further away than female dogs (6), other studies concluded the opposite (10, 18); some suggested that it depended on the neutered status (2, 8) while other studies concluded that the neutering status had no significant effect (14, 20). Contradicting results were also found regarding the impact of the body condition score (BCS) on the roaming behavior. BCS is an index used to visually assess a dog's body condition that ranges from one to five. Molloy et al. found that dogs with poor/fair BCS (<3) had larger core home ranges, potentially due to their need to feed themselves outside their homes (8). Yet, Pérez et al. stated that, except two outsiders, dogs with ideal BCS (i.e., 3) had larger home ranges than dogs with lower BCS (8, 13). On the other hand, findings were more consistent regarding food availability (10, 12, 27). A study on dog scavenging turtle nests highlighted that nest scavengers had lower metabolic intake of their daily food and significantly larger home ranges than non-nest scavengers (12). Similarly, studies in Brazil showed that higher stray dog density was associated with the proximity with potential sources of food, such as the university restaurants or commercial food outlets (10, 27). Another study in India concluded that groups of FRDD were more likely to be seen close to garbage sites (21). This suggests a substantial impact of feeding practices and quality and quantity of feed provided on FRDD movements.

Other predictors for roaming include environmental factors, such as the closeness of the owner's house to urban or rural settings (13), the type of setting (i.e., rural or urban) where the dog is living (10), or the season (mainly rainy vs. dry season) during which the roaming was measured (20, 22).

Variability in the findings between these studies could be explained by differences in the study design, in the analytical methods, or by the influence of site-specific factors (11). Study design differences include type of data collected (georeferenced capture sites or GPS collar locations), data collection period [from hours (19) to months (3)], or time interval between GPS fixes [from 15 s (5) to 30 min (1)]. Methodological differences can be found for home range size estimations, varying between minimum convex polygon (MCP) (6, 11, 13, 14, 18), the characteristic hull polygon (19), the time localized convex Hull (T-LoCoH) (16), the fixed kernel density distribution (1), and the biased random bridge method (BRB) (2–4). Dürr et al. compared several methods including the MCP, fixed kernel density distribution, T-LoCoH, and BRB and concluded that the BRB was more suitable for FRDD home range size estimation as it considers tracks and not only locations collected by the GPS devices (2).

Due to these differences between studies, comparative studies, performed on multiple countries, are needed to compare dog's behavior across countries and investigate whether differences in dog's roaming behavior can be predicted by the same factors worldwide or whether it depends on the site-specific context. The objectives of the current study are to compare FRDD demographics, management, and roaming behavior across four countries, namely, Chad, Guatemala, Indonesia, and Uganda, to identify and compare predictors for roaming in each of those countries.

## Methods

### Study Area

**Figure 1:**
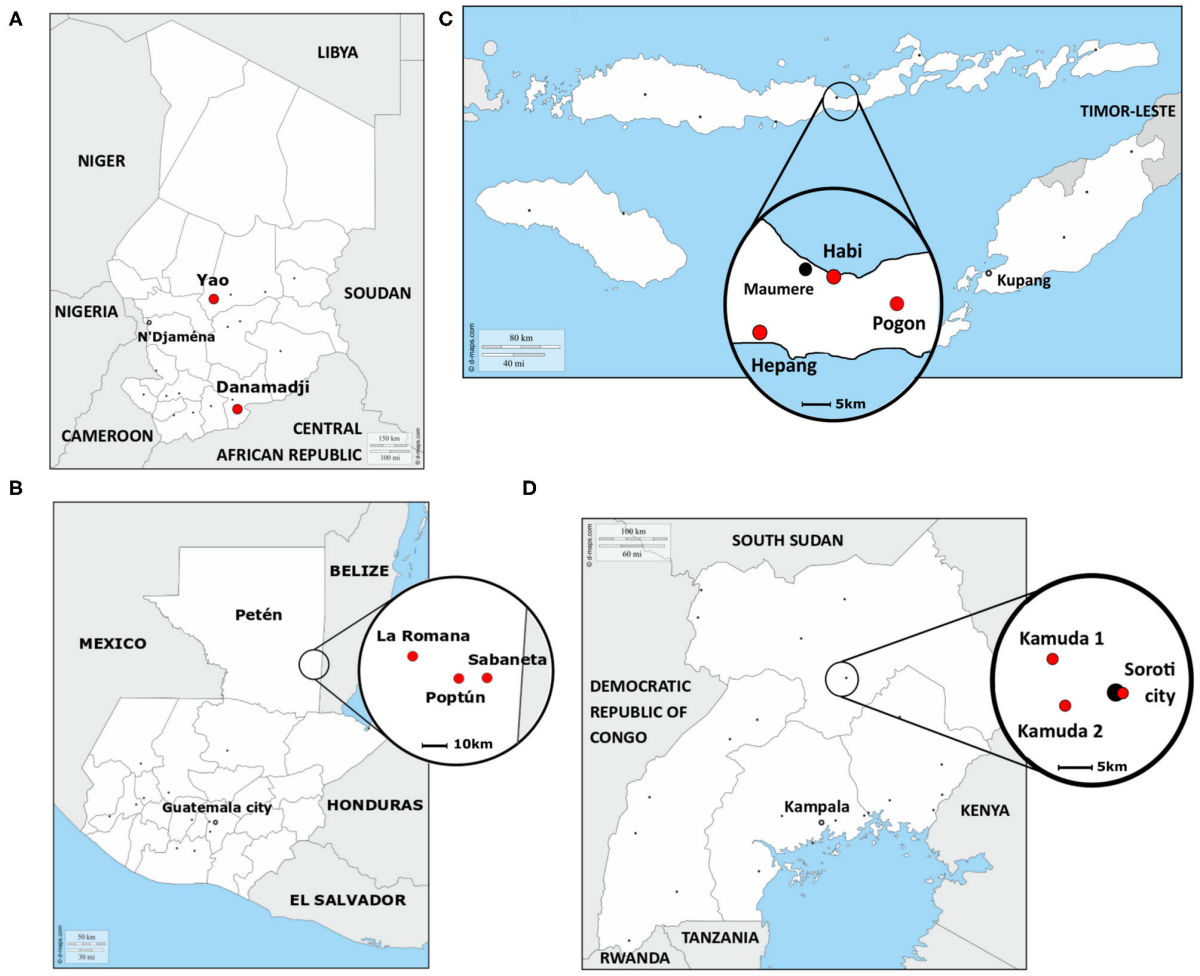
Localization of the eleven study sites in **(A)** Chad, **(B)** Guatemala, **(C)** Indonesia, and **(D)** Uganda. Red points: study sites, black points: main cities. The map of Guatemala was adapted from (28).

The study was performed in four countries, Chad, Guatemala, Indonesia, and Uganda (Figure 1), between January 2018 and February 2019, together with collaborative institutions from Universities and Government (Table 1). In each country, except Chad, three study sites were selected, two in a rural setting and one in an urban or semi-urban setting. In Chad, only two rural study sites were selected. The study site selection was achieved by the local team in synergy with ongoing research projects and based on the expected number of dogs living in each site (low, medium, and high—Table 1).

**Table 1:** Description of the study sites and data collection.

**Country**	**Study area**	**Village/town**	**Expected number of dogs**	**Study period**	**Localization**
Chad	Rural 1	NDakonon	Low	Jan/Feb 2018	Region: Moyen-Chari Department: Grande Sido District: Danamadji
	Rural 2	Sinetaye	Low		Region: Batha Department: Lake Fitri District: Yao
Guatemala	Rural 1	La Romana	Low	Mai/June 28	Department: Petén Municipality: Poptún
	Rural 2	Sabaneta	Medium		
	Urban	Poptún	High		
Indonesia	Rural 1	Pogon	Low	July 2018	Province: East Nusa Tenggara Island: Flores Regency: Sikka
	Rural 2	Hepang	Medium	August 2018	
	Semi-urban	Habi	High	July 2018	
Uganda	Rural 1	Kamuda 1	Low	Jan/Feb 2019	Region: Eastern Uganda District: Soroti
	Rural 2	Kamuda 2	Low		
	Semi-urban	Soroti municipality	High		

In both locations in Chad, the population is constituted of settled residents and mobile pastoralists. They are constituted of several families, living in concentration camps, while settled communities live in stable villages (29). Mobile pastoralists travel through transhumance routes during the wet season and stay near water points during the dry season. The main ethnic groups in Yao are Sara, Fulani, Goranes, and Arabs, in addition to other more local populations. Settled communities are mainly agriculturalists but often also keep livestock. Mobile pastoralists are livestock keeper, who sometimes cultivate food crops. Rabies is endemic in Chad with 20 to 90 persons dying from rabies every year according to the WHO (30). Rabies incidence data in the dog population are only available from the capital, N'Djaména (31, 32).

Petén department in Guatemala is scarcely populated with 69% Ladino (Spanish-speaking) and 30% Maya ethnicities (28). The Petén Department's main economic activity involves large plantations of African palm and cattle farms, yet the majority of the population's economic activity refers to subsistence agriculture, with a very small sector involved in tourism activities (33). The overall illiteracy rate is 22%, and nearly half of the population is living under the poverty rate (28). According to the WHO, two cases of human rabies were in Guatemala reported between 2013 and 2016, whereas no human rabies case was reported in Petén department since 1990 (30). From 2008 to 2014, five positive dogs were reported from the Petén Sur Oriental health area.

The Sikka Regency in Indonesia is mainly populated by Catholics of the ethnicities Sikka, Krowe, Tana Ai, Palue, and Lio (https://www.sikkakab.go.id/artikel-budaya). The main economic activity is agriculture (34). Nearly half of people graduated from elementary school and live with < US$52 per month (35). According to the WHO, an estimated 150 to 300 human cases of rabies occur in Indonesia every year (36). Flores Island, Indonesia, is endemic of canine rabies since 1998, and causes 15 human deaths annually (34, 37). During six months in 2019, 27 dogs were confirmed as rabies positive (https://mediaindonesia.com/nusantara/248367/wabah-rabies-menyebar-di-11-kecamatan-di-sikka).

Soroti district is located in Teso region in the eastern part of Uganda, mainly inhabited by the ethnic groups of Iteso and Kumam (38). The main economic activity is subsistence mixed farming with the majority being small holders engaging 68.3% of the households. Other sources of income for households include small-scale family businesses. Households in rural villages are scattered and grass thatched, while majority of houses in urban and peri-urban areas are built with walls, roof, and floors mainly constructed with temporal materials (39). Canine rabies is endemic in Uganda with an estimated 58 human dog bites per 100,000 persons (40) and 90 to 400 human deaths yearly (24). Global alliance for rabies control reported a total of 62 dog rabies positive samples between 2015 and 2020 in Uganda.

### Study Design

In each study site except Chad, a one-square-kilometer area was defined using Google Earth. Each dog whose owner was living in the defined areas was included in the study. In Chad, dogs were conveniently sampled in villages and nomad camps located in the two rural study sites. The exclusion criteria included the following categories: sick dogs, pregnant dogs, dogs younger than 4 months of age, dogs with necks too large to fit the GPS collar, absent dogs, or dogs of absent owner during all visits to the household (between one and three times).

Each dog owner was interviewed to collect data on dog demographics (sex, age, reproduction status, BCS) and management (role and origin of the dog, origin of the dog, human-mediated transportation within and outside the localization, feeding, confinement, and vaccination practices). All dogs of the household fulfilling the inclusion criteria were equipped with a georeferenced contact sensor, containing a GPS module, as used in previous studies (41). The GPS were set to record the dog position at a 1-min interval. The duration of the data collection was limited by the battery capacity and lasted in average 60 h. Dogs were vaccinated against rabies and/or dewormed. Due to practical reasons, it was performed at the same time than dog collaring in Chad, Guatemala, and Indonesia and during the removal of the collar in Uganda.

### Ethical Approval

Ethical clearance was sought separately in each country. In Chad, the National Bioethics Committee confirmed that no ethical approval was needed for the study according to their requirements; in Guatemala by the UVG's International Animal Care and Use Committee [Protocol No. I-2018(3)] and the Community Development Councils of the two rural sites, which included Maya Q'eqchi' communities (28); in Indonesia by the Animal Ethics Commission of the Faculty of Veterinary Medicine, Nusa Cendana University (Protocol KEH/FKH/NPEH/2019/009); and in Uganda by the Uganda National Council for Science and Technology (Protocol NS640).

The study was also introduced to the head of the households, or an adult person living in the same household, who gave written or oral consent, depending on the country.

### Home Range Estimation and Statistical Analysis

Data of dogs restricted at all times were excluded from the home range analysis. The GPS data were cleaned based on three criteria: horizontal dilution of precision (hdop), speed between two consecutive GPS fixes, and angle between a GPS fix and the previous and consecutive fixes. The hdop is a measure of accuracy automatically recorded by the devices for each GPS fix. GPS fixes with hdop higher than five were excluded, according to (42). The speed was calculated for each GPS fix by computing the distance in meters with the previous fix and dividing it by the difference in time between the two fixes. Both fixes were excluded if the speed exceeded 20 km/h, according to (2). The angle with the previous and consecutive points was calculated for each GPS fix, and the fixes having the 2.5% smallest angles were excluded, as small angles often suggest location errors (Supplementary Figure 1). Excluding 2.5% of the most acute angles was identified in providing good results in data cleaning (unpublished data).

The GPS locations, recorded under the World Geodetic System 1984, were converted to projected coordinate systems: UTM 34N for Chad, Guatemala Norte for Guatemala, UTM 51S for Indonesia, and UTM 36N for Uganda. Individual dog home range sizes were calculated using the BRB method (43), which has been earlier identified to suit to FRDD GPS data (2). The values for the BRB parameters were based on static tests with the GPS units (unpublished data). Lmin corresponds to the minimum distance between successive fixes to identify a movement (i.e., when the distance between two fixes is lower than Lmin, the function considers that the animal is not moving) and was set at 38 m (44). Hmin is a smoothing parameter depicting the minimum uncertainty over the location of the dog and was set at 17 m (44). The size of the 50% (core home range) and 95% isopleth (extended home range) were extracted for each dog. Home range sizes were compared between countries and between study sites within countries using the Wilcoxon–Mann–Whitney test.

Linear regression models were developed, for each country separately, to identify predictors for core and extended home range size. The factors investigated were sex, age (as categorical variable), BCS, and role (i.e., guardian dog, hunting dog, shepherd dog, or dog raised for meat) of the dog, time the dog is allowed to roam freely (free-roaming time, FRT), and study site (Table 2). The role of the dog was included as binary dummy variables since each dog can have multiple roles. Being a pet was not investigated because we considered the variable to be too subjective and country-dependent to provide comparable results between countries. Correlation between variables was assessed using a Chi-squared test and BCS was excluded from the analysis in Chad (associated with sex, being a shepherd dog, and FRT); FRT was excluded in Guatemala (associated with BCS) and BCS was excluded in Indonesia (associated with being a guardian dog). Dogs restricted ceaselessly and dogs whose collar recorded <24 h were excluded from the regression analysis because the home range size would not reflect the dog's normal and representative roaming behavior.

**Table 2:** Explanatory variables investigated in the country-specific regression models.

**Variable**	**Type of variable**	**Levels**	**Models where the variable was included**
Sex	Binary	0: male (baseline)1: female	All countries
Age	Binary	Adult: ≤ 12 months and <72 months Young: <12 months Senior: ≥72 months	Indonesia and Uganda
BCS	Ordinal	Level 1: very thin Level 2: underweight Level 3: ideal body score (baseline) Level 4: overweight Level 5: obese	Guatemala and Uganda
Being a guardian dog	Binary	0: no 1: yes	Guatemala and Indonesia
Being a shepherd dog	Binary	0: no 1: yes	Chad and Uganda
Being a hunting dog	Binary	0: no 1: yes	Chad and Guatemala
Being raised for meat	Binary	0: no 1: yes	Indonesia
Free-roaming time (FRT)	Categorical	Always: never confined (baseline) Day only: confined during the night Night only: confined during the day Sometimes: confined during a shorter period than day or the night	Indonesia and Uganda
Study site	Categorical	Name of the study site	All countries

The selection of the best regression models was based on Akaike Information Criterion (AIC). For each country separately, sets with all possible combinations of the five (Chad and Guatemala) and six (Indonesia and Uganda) factors were generated (Table 2). This led to 31 and 63 combinations for countries with five and six investigated factors, respectively. These sets of factors were used as explanatory variables in linear regression models with the logarithm of the size of the 50% and 95% isopleth, respectively, as outcome variable. The AIC of each model was extracted and compared to the AIC of the best model (i.e., model with the lowest AIC) per country and outcome. Models with a difference in AIC of less than two were selected and considered as “best models.” The regression coefficient, the coefficient 95% confident intervals, and the *p*-value of these best models were extracted and compared between countries. If a variable was selected in several “best models,” the coefficient and *p*-value of the model having the lowest AIC were considered for comparison. The normality of the residuals was visually assessed for each best model. To assess the impact of short recording periods of some dogs on the identification of home range predictors, a sensitivity analysis was performed by applying the same regression analyses to dogs whose collar recorded data for at least 48 h.

Descriptive statistics of the questionnaire data were performed using R software and the packages *dplyr, ggplot2, gridExtra*, and *ggsignif* . Home ranges were computed using the packages *adehabitatHR* and *adehabitatLT*.

## Results

### Dog Demography

In this study, we collared 773 dogs including 106 in Chad, 303 in Guatemala, 217 in Indonesia, and 149 in Uganda. The dog population was constituted of a majority of male dogs in Chad (75%) and Guatemala (65%) and female dogs in Indonesia (63%). In Uganda, the sex ratio was balanced at the country level (50% of male and female dogs (Table 3). Younger dogs were found in Indonesia compared to Chad and Uganda. No reliable age data were available for Danamadji (Chad) and all the three Guatemalan study sites. The median BCS was underweight (i.e., 2) or ideal (i.e., 3) depending on the study site (Table 3). The proportion of neutered dogs was below 5% in all study sites except one with a very small sample size (Kamuda 2–n=8). Nearly all neutered dogs were males (92%−23/25). The two neutered females were living in Uganda.

**Table 3:** Demographics of the dogs collared in eleven study sites in Chad, Guatemala, Indonesia, and Uganda.

	**Chad**		**Guatemala**			**Indonesia**			**Uganda**		
	**Danamadji**	**Yao**	**La Romana**	**Sabaneta**	**Poptún**	**Pogon**	**Hepang**	**Habi**	**Kamuda 1**	**Kamuda 2**	**Soroti city**
Sample size	52	54	61	125	117	52	65	100	17	8	124
Sex ratioMale:female	1.4: 1	9.8: 1	1.7: 1	2.4: 1	1.5: 1	0.5: 1	0.8: 1	0.5: 1	1.1: 1	7: 1	0.9: 1
Age (months)*Median (1^*st*^-3^*rd*^ quartile)*	-	36 (24–60)	-	-	-	12 (7–30)	12 (6–15)	9.5 (6–24)	24 (12–36)	48 (18–69)	30 (15–56.2)
Body conditioning score*Median (1^*st*^-3^*rd*^ quartile)*	2 (2–2)	3 (3–3)	3 (2–3)	2 (2–3)	3 (2–3)	2 (2–3)	2 (2–2)	3 (3–3)	3 (2.75–3)	3 (3–3)	3 (3–3)
Percentage of neutered dogs (n/N)	4 (2/52)	0 (0/54)	3 (2/58)	4.9 (6/122)	0.9 (1/116)	4 (2/51)	2 (1/63)	3 (3/98)	0 (0/17)	4/8	3.2 (4/124)

### Dog Management

**Figure 2:**
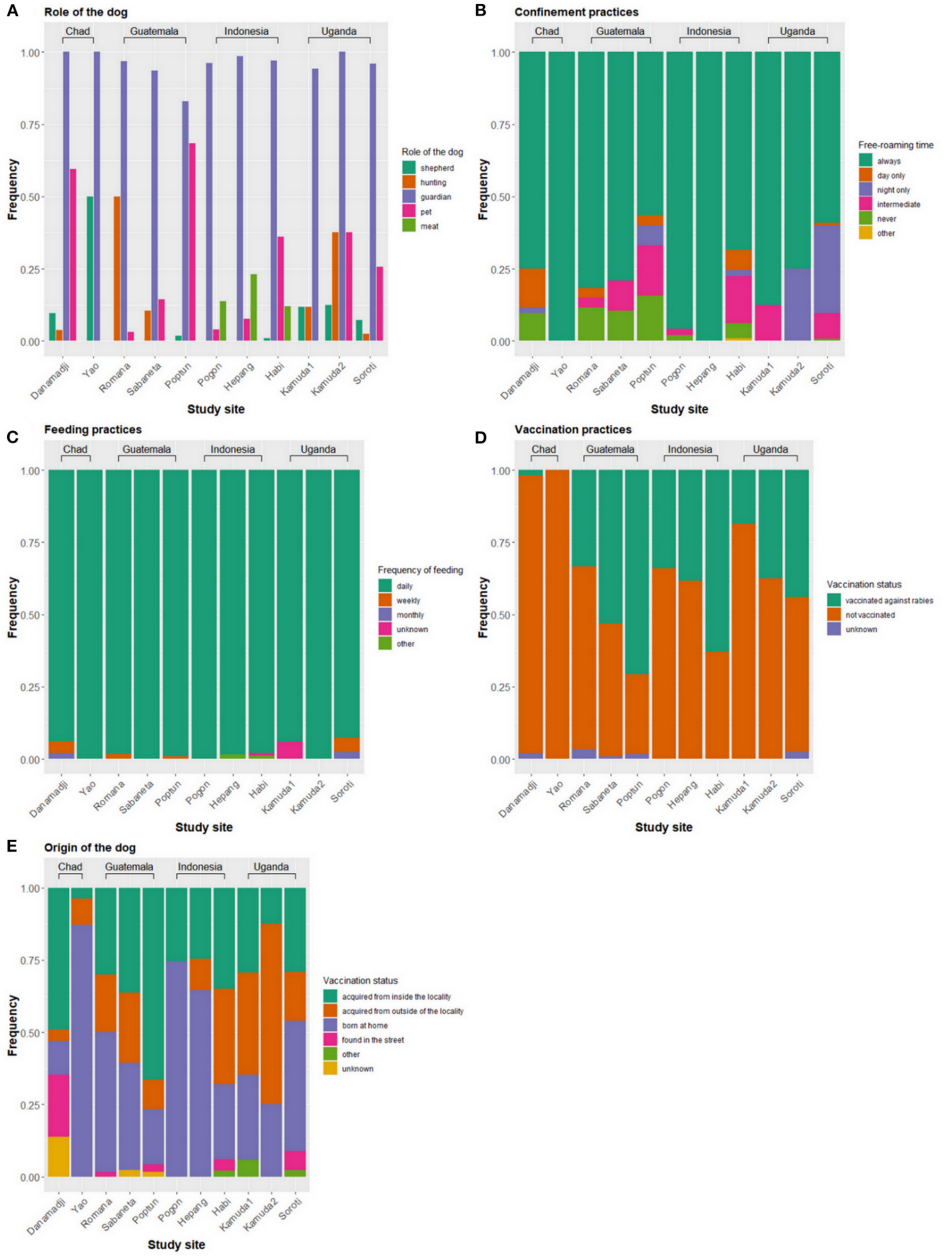
Management practices of domestic dogs across eleven study sites in Chad, Guatemala, Indonesia, and Uganda. **(A)** Role of the dog, **(B)** confinement practices, **(C)** feeding practices, **(D)** vaccination practices, and **(E)** origin of the dogs.

In all study sites, nearly all dogs were primarily kept for protection purposes (Figure 2A). Dogs were more frequently used for shepherding in Chad than in the other study sites. Hunting dogs were more common in Guatemala. Several dogs were raised for their meat in Indonesia. Dog meat consumption was also reported in Danamadji (Chad) but not systematically recorded in the questionnaire. Confinement practices varied between study sites, but at least half of the dogs were allowed to always roam freely (Figure 2B). In all countries with urban and rural sites, dogs that always roam freely were more often found in rural areas (*p* <0.001 in Guatemala and Indonesia, *p* = 0.058 in Uganda). Nearly all dogs were fed by their owners on a daily basis (Figure 2C). The dogs were mostly fed with leftovers, whose composition varied between countries and study sites. For example, owners reported feeding the dogs with milk in Yao (Chad), corn tortillas in Guatemala, and rice, fish, tarot, and corn in Indonesia. Except for Poptún (Guatemala) and Habi (Indonesia), most of the dogs were not vaccinated against rabies before our study (Figure 2D). The proportion of dogs that had already experienced a vaccination prior to our study was higher in urban/semi-urban than rural sites in all three countries, although not always statistically different (*p* <0.001 in Guatemala and Indonesia and *p* = 0.11 in Uganda).

The percentage of owners who reported moving with their dogs outside of the locality (village or town) was low, with 13% (12/92) in Chad, 7% (13/187) in Guatemala, 2% (3/153) in Indonesia, and 2% (2/81) in Uganda. The percentage of owners who reported moving with their dogs inside the locality was 72% (67/92) in Chad, 63% (117/187) in Guatemala, 12% (18/153) in Indonesia, and 2% (2/81) in Uganda.

In Chad, few dogs were acquired from outside of the locality and owners reported that those came from neighboring villages and camps (Figure 2E). In Guatemala, the percentage of dogs acquired from outside of the locality was higher and owners reported acquisitions from faraway places. Two dogs had come from Belize, a neighboring country, and four dogs from other departments in Guatemala (two from Izabal and two from Zacapa). More dogs were acquired from outside of the locality in urban/semi-urban area (i.e., Habi) in Indonesia and rural areas (i.e., Kamuda 1 and 2) in Uganda. Because of questionnaire modifications, place of origin for dogs acquired from outside of the locality in Indonesia and Uganda is not available.

In Chad, 29% of the dogs belonged to nomad pastoralists, while the rest of the dogs belonged to villagers (47%) or former nomads who settled definitively (24%). More dogs were owned by nomad pastoralists in Yao (74%) than in Danamadji (8%).

### Dog Home Range

After data cleaning, GPS data were available from 100 dogs in Chad (47 in Danamadji and 53 in Yao), 254 dogs in Guatemala (50 in La Romana, 113 in Sabaneta, and 91 in Poptún), 149 in Indonesia (36 in Pogon, 40 in Hepang, and 73 in Habi), and 95 dogs in Uganda (14 in Kamuda 1, 6 in Kamuda 2, and 75 in Soroti). In total, data from 77 dogs had to be removed because the data collection period was <24 h or <50 GPS fixes have been recorded and data from 10 dogs had to be removed because of missing factor data (Supplementary Table 1). The data collection period 2.5 and 97.5 percentiles of the dogs used to estimate home range size ranged between 28.6 and 113.3 (median 60.3) h, with 114–5,740 (median = 1,357) GPS fixes recorded per dog.

The core home range size (50% isopleth) 95% range was between 0.17 and 0.92 ha (median: 0.30 ha) in Chad, 0.17 and 1.1 ha (median: 0.33 ha) in Guatemala, 0.20 and 0.61 ha (median: 0.30 ha) in Indonesia, and 0.15 and 0.72 ha (median: 0.25 ha) in Uganda. The extended home range size (95% isopleth) 2.5 and 97.5 percentiles ranged between 1.1 and 103 ha in Chad (median: 7.7 ha), 1.5 and 27.5 ha in (median: 5.7 ha) Guatemala, 1.6 and 26.5 ha (median: 5.6 ha) in Indonesia, and 1.3 and 19.1 ha (median: 5.7 ha) in Uganda. Core and extended home range size distribution were right-skewed in all study site countries (Supplementary Figure 2).

**Figure 3:**
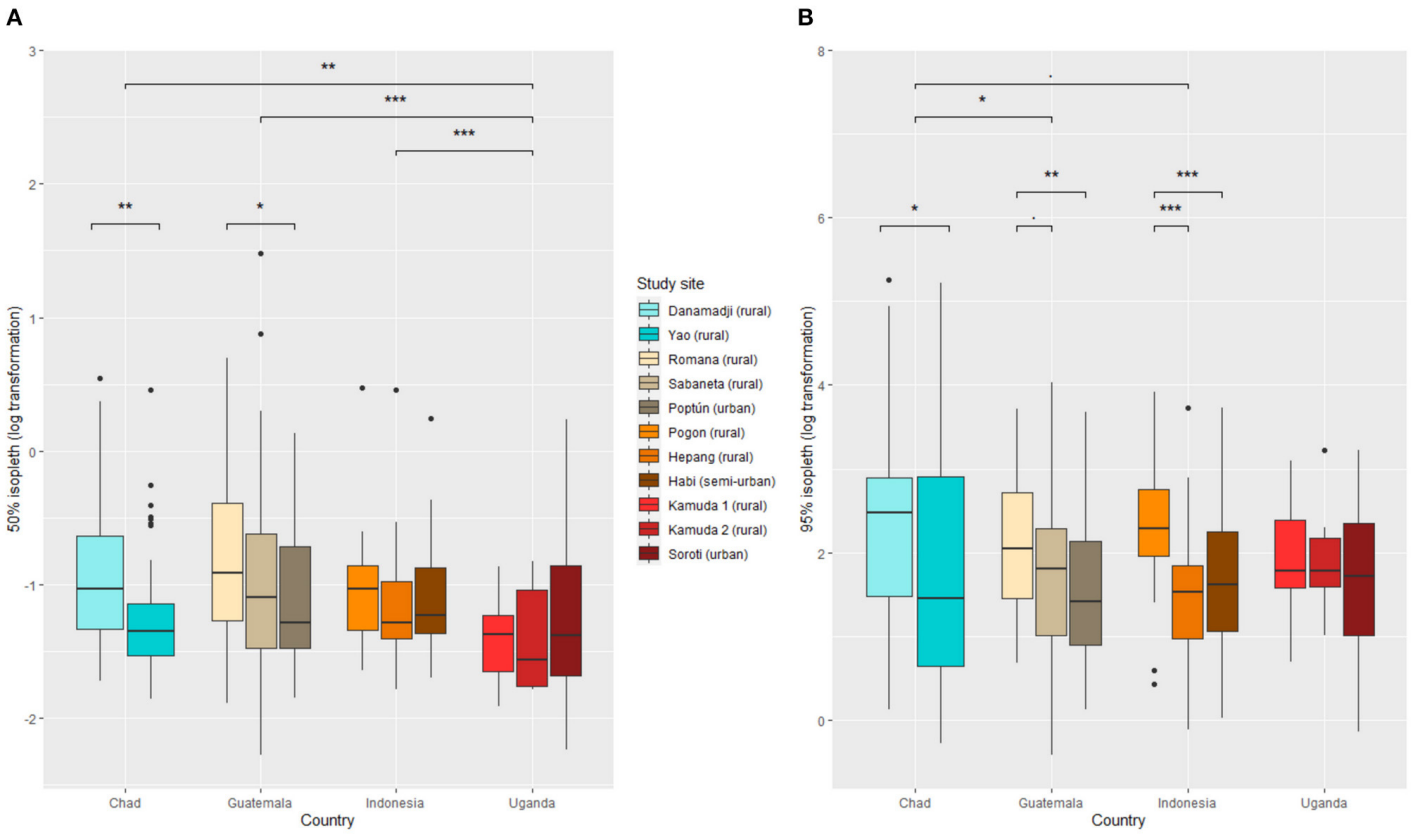
Dog home range size across eleven study sites in Chad, Guatemala, Indonesia, and Uganda. **(A)** 50% isopleth and **(B)** 95% isopleth. The three (respectively two) top bars represent the significance level between countries for the 50% isopleth (respectively 95% isopleth). The two (respectively five) other bars represent the significance level between study sites within countries for the 50% isopleth (respectively 95% isopleth). Significance codes: 0.001= ^***^ , 0.01= ^**^ , 0.05= ^*^ , 0.1=.

Home range sizes varied between countries and study sites (Figure 3). In Guatemala, dogs living in urban setting (i.e., Poptún) tended to have smaller home ranges than dogs living in rural settings, but this was not confirmed in Indonesia and Uganda.

### Explanatory Factors for Dog Home Range Size

**Figure 4:**
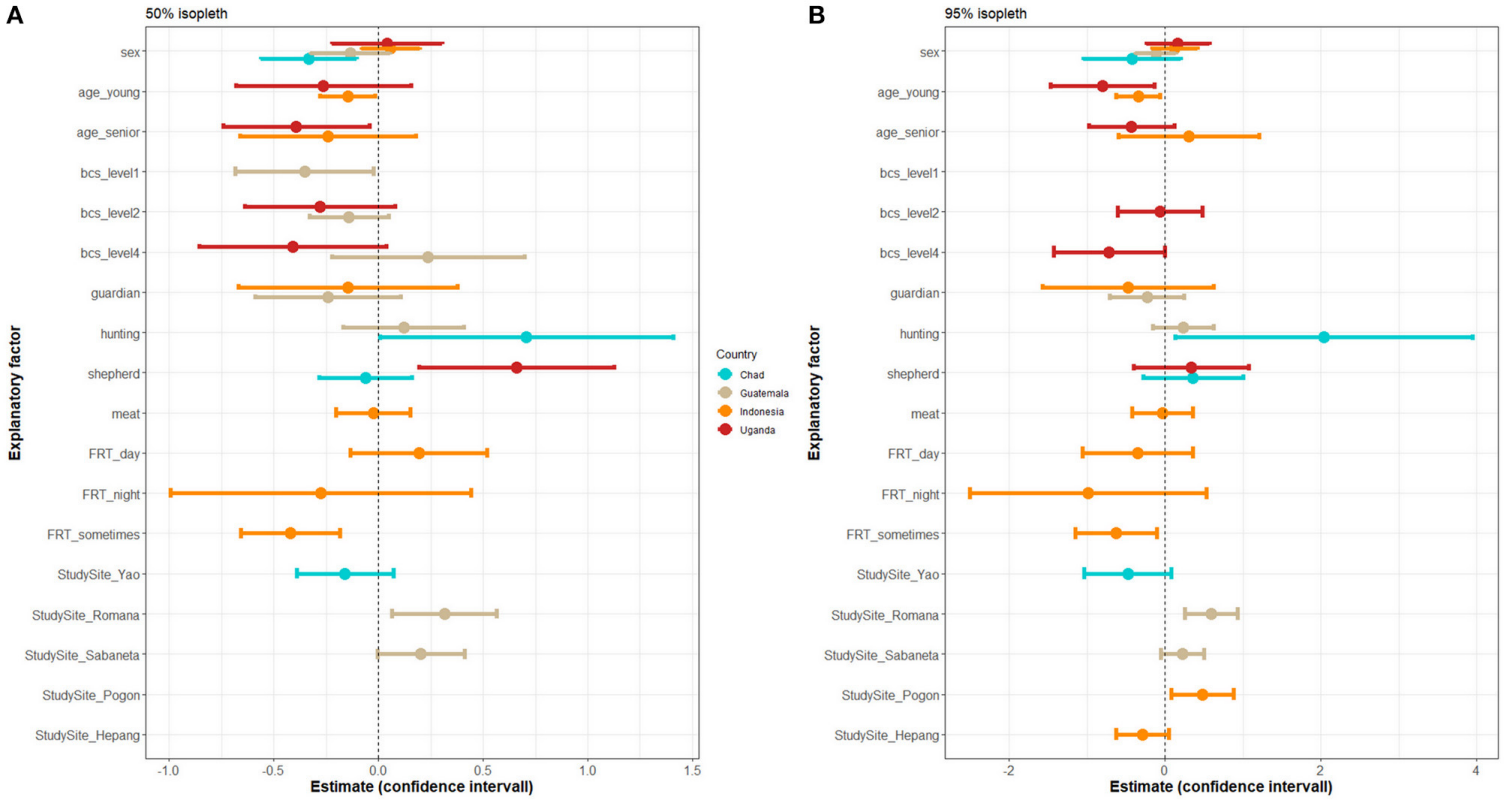
Linear regression model estimates and confidence intervals of each explanatory factor selected in the best models on the outcomes: **(A)** logarithm of 50% isopleth HR size and **(B)** logarithm of 95% isopleth HR size. A set of “best models” was selected separately for each country. Sex [binary−0:male (baseline), 1: female] was investigated in the four countries; age (discrete, age in months) was investigated in Indonesia and Uganda; body condition score (BCS, ordinal—ranging from 1 to 5) was investigated all four countries; being a guardian dog (binary) was investigated in all four countries; being a shepherd dogs (binary) was investigated in all four countries; being a hunting dog (binary) was investigated in Chad, Guatemala, and Uganda; being raised for meat (binary) was investigated in Indonesia only; and free-roaming time (FRT): the time when the dog was unrestricted [categorical, level: always (baseline), during the day, during the night, intermediary] was investigated in all four countries.

The set of predictors for the core and extended home ranges varied between countries (Figure 4 and Supplementary Tables 1, 2 for details). Sex was a predictor of the home range size in Chad only, where male dogs tended to have larger core home ranges. Younger dogs had smaller home ranges in both countries where reliable age data was available (Uganda and Indonesia). Senior dogs had smaller home ranges in Uganda. Dogs with poor BCS tended to have smaller core home ranges in Guatemala.

Hunting dogs in Chad had larger home ranges than dogs not used for this purpose, whereas this factor did not significantly influence the home range size in Guatemala. Being a shepherd dog was associated with larger core, but not extended, home ranges in Uganda, yet it had no significant impact in Chad. Being a guardian dog or being raised for meat was not found to have an impact on the home range size. The time when the dog was allowed to roam freely had an impact in Indonesia only, where dogs confined during longer periods of time (i.e., “sometimes”) had smaller home ranges than never confined dogs.

The study site was selected in the regression models as an explanatory variable in Chad (but not significant), Guatemala (as predictor of both core and extended home ranges), and Indonesia (as predictor of the extended home range), but not in Uganda. In Guatemala, dogs living in urban/semi-urban setting (i.e., Poptún) had smaller home ranges than dogs living in rural settings (i.e., La Romana and Sabaneta). In Indonesia, dogs living in the urban/semi-urban setting (i.e., Habi) had smaller home ranges than dogs living in one rural study site (i.e., Pogon) but tended to have larger home ranges than dogs living in the other rural study site (i.e., Hepang).

The results of the model restricted to dogs collared for at least 48 h differed mainly for age and BCS (Supplementary Table 3). For age and BCS, some significances changed using the threshold of error I of 0.05; however, the trends remained. Being a very thin dog (*p*-value of 0.04 in the main model and 0.1 in the restricted model) and being a senior dog (*p*-value of 0.03 in the main model and 0.06 in the restricted model) remained negatively associated with the core home range, while having a high BCS (*p*-value of 0.08 in the main model and 0.03 in the restricted model) newly showed a significant negative association. Similarly, being a young dog (*p*-value of 0.02 in the main model and 0.05 in the restricted model) remained negatively associated with the extended home range, while having a BCS of 4 (*p*-value of 0.05 in the main model and 0.03 in the restricted model) newly showed significant negatively association.

## Discussion

We here present a large comparative study on the demography, management, and roaming behavior of 773 FRDD from four different countries around the world. To our knowledge, this is the first study that compares such characteristics of FRDD populations across three continents. According to the study outcomes, dog demographic, management, and roaming behavior varied between study sites and countries, under the here examined conditions. Although we could identify some predictors for the dog home range size, namely, sex, age, BCS, role of the dog, and time when the dog is not restrained, these factors were only significant for some sites, but not for others.

**Figure 5:**
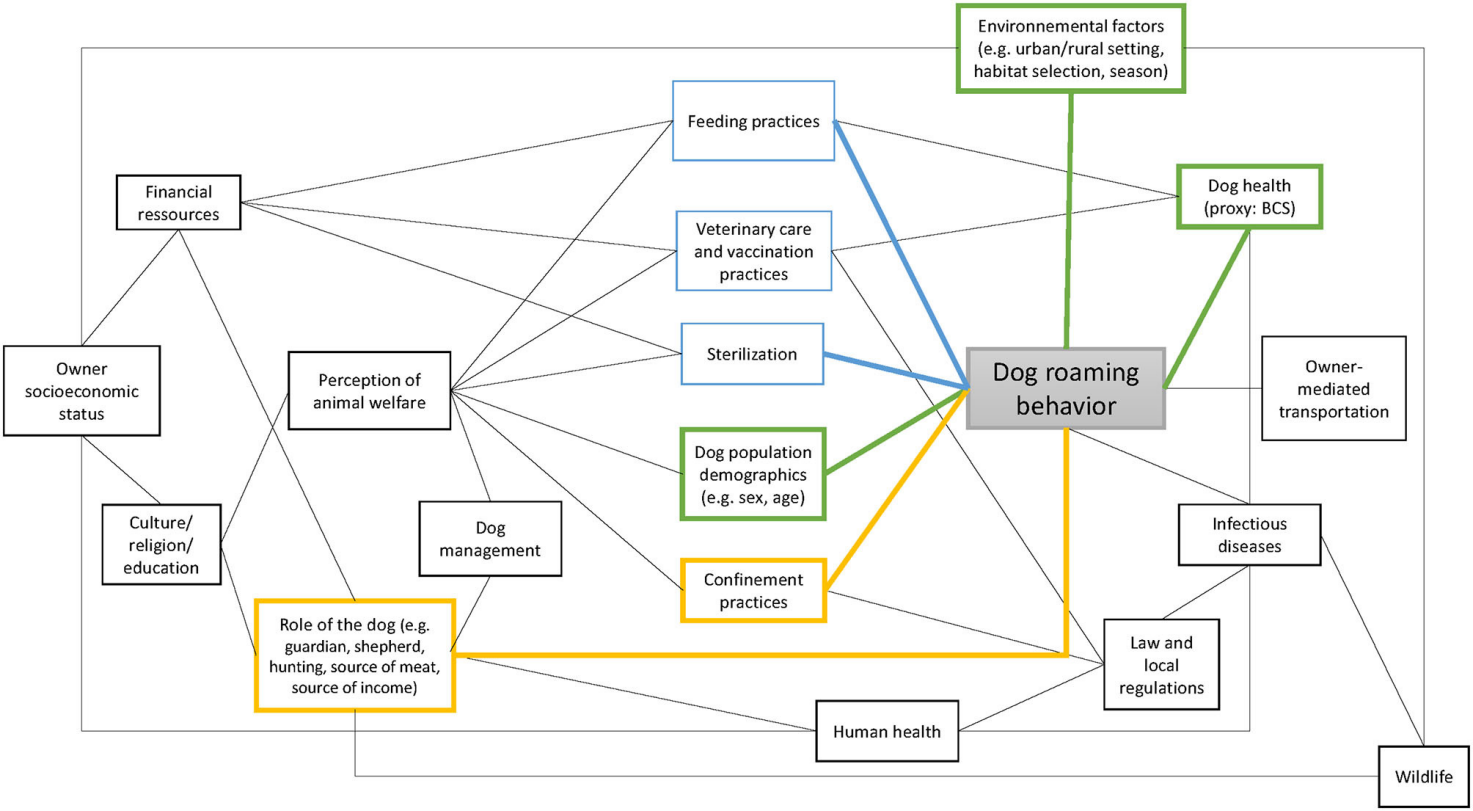
Graph illustrating potential factors influencing the dog roaming behavior. The black links represent potential relationships. The blue links represent the factors associated with dog roaming behavior according to the literature, the yellow links represent the factors associated with dog roaming behavior in this study, and the green links represent the factors associated with dog roaming behavior in both the current study and the literature. This graph is not meant to be exhaustive.

The FRDD population is complex given that they are neither fully supervised pets nor wild animals (such as wild dogs or dingoes). Also, FRDD are typically strongly bound to their owners and therefore follow them—depending on the settings—which makes them different to livestock. An attempt at illustrating their complexity of behavior is presented in Figure 5. Our study showed how the interrelatedness of these components impedes identification of universal predictors for roaming patterns in FRDD.

Yao, in the center of Chad, was the study with the highest proportion of male dogs. It was also the study site with the highest proportion of dogs owned by nomad pastoralists, living in mobile camps (29). In these communities, guardian and shepherd dogs are of high importance to protect the people and their livestock against wild animals (hyenas and jackals). It was suggested during the interviews that owners would preferentially keep intact male dogs because of their larger size, which would be an asset to chase wild animals off. In addition, some owners reported that, because of the high number of intact male dogs, having female dogs could cause fights between male dogs when the female is in estrus. Without reporting the reason, owning female dogs was also reported to cause problems by dog owners in completely different settings in Chile (45). Indonesia was the only country where the proportion of female dogs was higher than the proportion of male dogs. This might result from dog meat consumption, which is common in Flores Island, especially during traditional ceremonies (34). Keeping female dogs is favorable for breeding and financial purposes, since dogs are also bred for commercial purposes. Dog breeding and consumption might also have influenced the dog's age distribution. Dogs in Indonesia are substantially younger compared to the other countries, where good-quality age data was available. Slaughtering adult dogs and foster reproduction are expected to have lowered the age average of the population. Both examples highlight the relationship between the owners' socioeconomic status, the role of the dog, the dog management, and its impact on the dog population demography (Figure 5).

A substantial proportion of owners reported the origin of their dogs being inside or outside of the locality (Figure 2E), which suggests owner-mediated transportation on short or long distances. While short-range transportation of dogs may cause an increase of local spread of canine infectious diseases, such as rabies, large-range transportation was found to be particularly critical because of its role in rabies sustainability at the country level (46). In our study, dogs originating from outside the locality were more often found in rural study sites in Guatemala and Uganda, and in the semi-urban site in Indonesia. In Guatemala, a few dogs originated from far away, such as from other departments or even Belize (a neighboring country). Similar long-range movements were previously described in Chile (47). The investigation of human-mediated dog movements is also of particular interest for nomad pastoralists in Yao. Despite that dog owners in Yao reported acquiring the dogs from neighboring villages or camps, nomad pastoralists are involved in long-range movements during transhumance (29) and therefore may be involved in potential spread of infectious diseases via their dogs over larger regions. However, during the study period, the GPS data did not provide any evidence of long-range movements.

On the other side, short-distance transportations are of particular interest for the current study because the dog home range is impacted by short-distance transportation (and therefore predictors for the home range size). By excluding GPS fixes based on speed, we aimed at excluding transportation by car before calculating the home range size. Yet still, we cannot differentiate dog movements motivated by the owner or the dog itself. In Guatemala and Chad, it was common for people to move with their dogs inside the locality (72% and 63% of the owners respectively), while it happened less frequently in Indonesia and Uganda (12% and 2% of the owners respectively). This could explain why the predictors for roaming vary between countries. If the owner has a larger impact on the dog movements in some countries, it could have lowered the impact of other factors. It also raises the question of home range definition for FRDD. The home range is the area commonly used for normal, typically daily activities. Although a unique hunting trip might not be defined as a normal activity, it can well be counted as such if the same trip happens frequently. For example, shepherd dogs might follow their owners to the grazing areas every day or guardian dogs their owners in the village or to the market. Because of FRDD proximity with humans, dog movements cannot be completely separated from human movements (Figure 5), which need to be considered when interpreting FRDD home range.

The home range sizes computed in the four countries were similar to each other and to findings from previous studies using the BRB method to calculate the home range size (3, 8, 22). Previous study on FRDD in Brazil suggests that the home range size is larger in rural compared to urban settings (10). We could only reproduce this finding in Guatemala, the country with the most urbanized study site (Poptun). In Indonesia, dogs living in Habi (the semi-urban study site) had a smaller home range compared to one rural site, Pogon, but not to the other, Hepang. This might be due to the village setting in Hepang, where houses were concentrated along the main road and distances between households were shorter than in Habi. In Uganda, the number of the dogs observed in the two rural areas might have been too small to detect a difference. As the study site was found to have an impact on dog roaming behavior, it would be of interest to investigate study site-specific factors such as road distribution, rural or urban setting, or human and dog density. In our study, we were not able to investigate such parameters independently, because of their high correlation among each other (e.g., human density and road structure). More studies are needed to confirm the hypothesis that dog home ranges in urban settings are smaller than in rural settings, and to potentially identify the underlying reason.

Sex and age were found to influence the home range size in some study sites. Sex was a predictor for roaming in Chad only, with female dogs having a smaller core home range compared to males. This result reflects the inconsistency of the influence of sex on roaming behavior found in the literature (6, 8, 10, 14, 18–20, 22) and supports the hypothesis that sex is not a clear influencing factor. The influence of age on the home range size was only investigated in Uganda and Indonesia. Senior dogs had smaller home ranges in Uganda, which could be explained by lower activity levels in older dogs. However, this finding was not replicated in Indonesia. Younger dogs had smaller home ranges in both study countries. This might be due to young dogs staying close to their mother (dogs were collared from 4 months of age). This result contradicts previous studies from Australia, where age was not found to be associated with the roaming behavior (8, 20, 22) and a study in Chile where young dogs were traveling further away from home (13).

The BCS was investigated in Guatemala and Uganda and was identified as being an influencing factor on the home range size. Dogs with poor BCS (1: very thin) had significantly smaller core home ranges in Guatemala only. Contradictory results are found in the literature, where dogs with lower BCS (1–2: very thin to underweight) had larger home ranges (8, 13). They hypothesized that these dogs might have limited access to food sources at home and roam further for scavenging (8). This is supported by a study in Mexico, where dogs scavenging turtle nests had lower food metabolic intake provided by their owners and larger activity ranges (12). In Uganda, dogs with BCS of 4 tend to have smaller home ranges compared to dogs with ideal BCS, a finding that could not be reproduced in Guatemala. These examples highlight the relationship between feeding practices, dog's health (approximated by BCS), and roaming behavior (Figure 5). We could not directly investigate the association between feeding practices and roaming behavior since the frequency of feeding and the main source of food provided were similar for most dogs (Figure 2C) and we did not collect data on the quantity of food provided to the dogs. Nearly all owners reported feeding their dogs on a daily basis with leftovers. However, social desirability bias may have impacted our data and the actual feeding frequency might be lower than stated, which would explain the low dogs' BCS in some of the study sites. Observations during previous studies in the same villages in Guatemala suggest that dogs were not fed daily when there were no leftovers (unpublished data).

Being a shepherd dog, being a hunting dog, and time when the dog was not restricted are three dog management factors associated with dog home range in some of the countries investigated. These three factors are directly controlled by the owner and therefore depend on the owner's occupation, cultural background, and perception of animal welfare (Figure 5). In some communities, free-roaming is considered to have a positive impact on dog well-being (45), while in other regions free roaming is primarily considered as a negative impact for the potential transmission of infectious diseases, such as rabies (48). Differences between countries, regarding the impact of the dog's role, might again be caused by cultural and environmental characteristics (e.g., weather, season, land use). The tasks of a guardian, shepherd, or hunting dog might differ from one country to another. For example, shepherd dog roaming behavior was described in Australia and showed that dogs spent most of their time with livestock (1). Therefore, movements of shepherd dogs depend on the type of livestock, the husbandry practices, and food accessibility for livestock. In Chad, livestock—and together with them the shepherd dogs—could cover long distances to access grazing areas (49). Similarly, hunting dog movements are impacted by hunting practices, which are related to the local context and culture of dog owners (e.g., type of prey, hunting format, or frequency of hunting trips) (Figure 5).

In this study, the GPS collars collected data during an average duration of 60 h, limited by the battery capacity. We explored whether excluding of dogs with <48 h of data collection, that is, those dogs with <2 daytime periods recorded, displayed differences in the identification of factors influencing the home range size. While some differences were found, the same trends identified by the full dataset remained (Supplementary Table 3). However, it is likely that the dog home range would have changed if we had collected GPS data over a longer period (e.g., several weeks or months). The core home range is likely to be more stable over time than the extended home range, since the latter is more prone to single trips of short duration (3). However, studies investigating required monitoring period to accurately represent the dog home range are currently lacking.

Another limitation of this study is the potential collinearity of the independent variables within the regression models. For example, young dogs might be categorized with a low BCS, even if their body condition is normal for their age. Using analytical methods that consider the dependency between variables might help to overcome this issue.

In this study, we highlighted similarities and differences in the demography, management, and roaming behavior of FRDD living in Chad, Guatemala, Indonesia, and Uganda. We identified some predictors (sex, age, BCS, being a hunting or shepherd dog, time when the dog is allowed to roam freely) for home range size but acknowledge that dog roaming behavior is complex and intrinsically related to its owner (socioeconomic status and transportation habits) and the environment of the locality. Therefore, FRDD cannot be all put under the same umbrella and a deeper understanding of the local culture and dog–human relationship is essential when dealing with FRDD issues, whether rabies control is concerned or not.

## Data Availability Statement

The raw data supporting the conclusions of this article will be made available by the authors, without undue reservation.

## Ethics Statement

The animal study was reviewed and approved by the Universidad del Valle de Guatemala's International Animal Care and Use Committee (Protocol No. 175-04-2018) in Guatemala; the Animal Ethics Commission of the Faculty of Veterinary Medicine, Nusa Cendana University (Protocol EH/FKH/NPEH/2019/009) in Indonesia; and the Uganda National Council for Science and Technology (Protocol NS640) in Uganda. Written or oral informed consent were obtained from all owners, in accordance with the country-specific regulations.

## Author Contributions

CW, MFA, DA, MB-G, TO, EW, SH, and SD: designed the research. CW, TO, EW, PB, GA, MM, VB, AL, EM, FM, AN, and PR: performed the data collection. LC provided data on the GPS accuracy. CW analyzed data. SD assisted with data analysis. CW wrote the paper with contributions from SD. All authors read and approved the final version of the paper.

## Conflict of Interest

The authors declare that the research was conducted in the absence of any commercial or financial relationships that could be construed as a potential conflict of interest.
