# Supplementary material for: Comparative Study of Free-Roaming Domestic Dog Management and Roaming Behavior Across Four Countries: Chad, Guatemala, Indonesia, and Uganda
Source: Front Vet Sci. 2021 Mar 4;8:617900. doi: 10.3389/fvets.2021.617900 (PMC7970034; doi:10.3389/fvets.2021.617900)
Supplement: Supplementary file 1 [file Data_Sheet_1.docx]

Supplementary Material

**
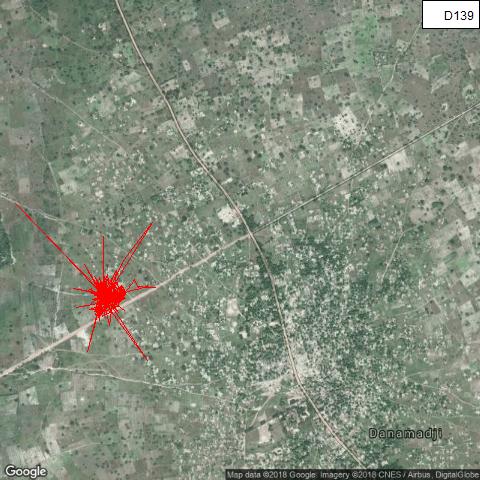
**

**
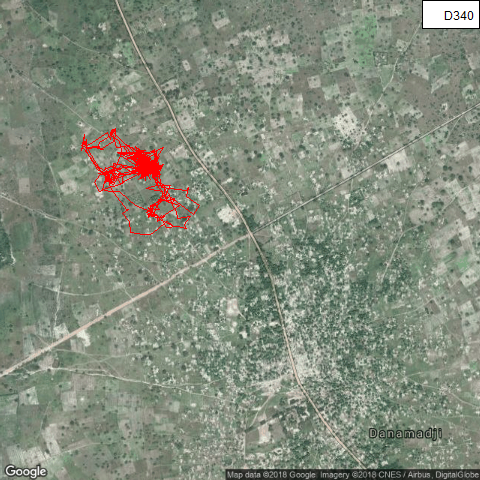
**

A

B

**Supplementary Figure 1**. Examples of two dog trajectories in Danamadji, January 2018 with (A) small angles suggesting location errors and (B) visually normal trajectories. The maps were generated using sp, adehabitatHR and RgoogleMaps packages. Source of the maps: Google Maps.

**
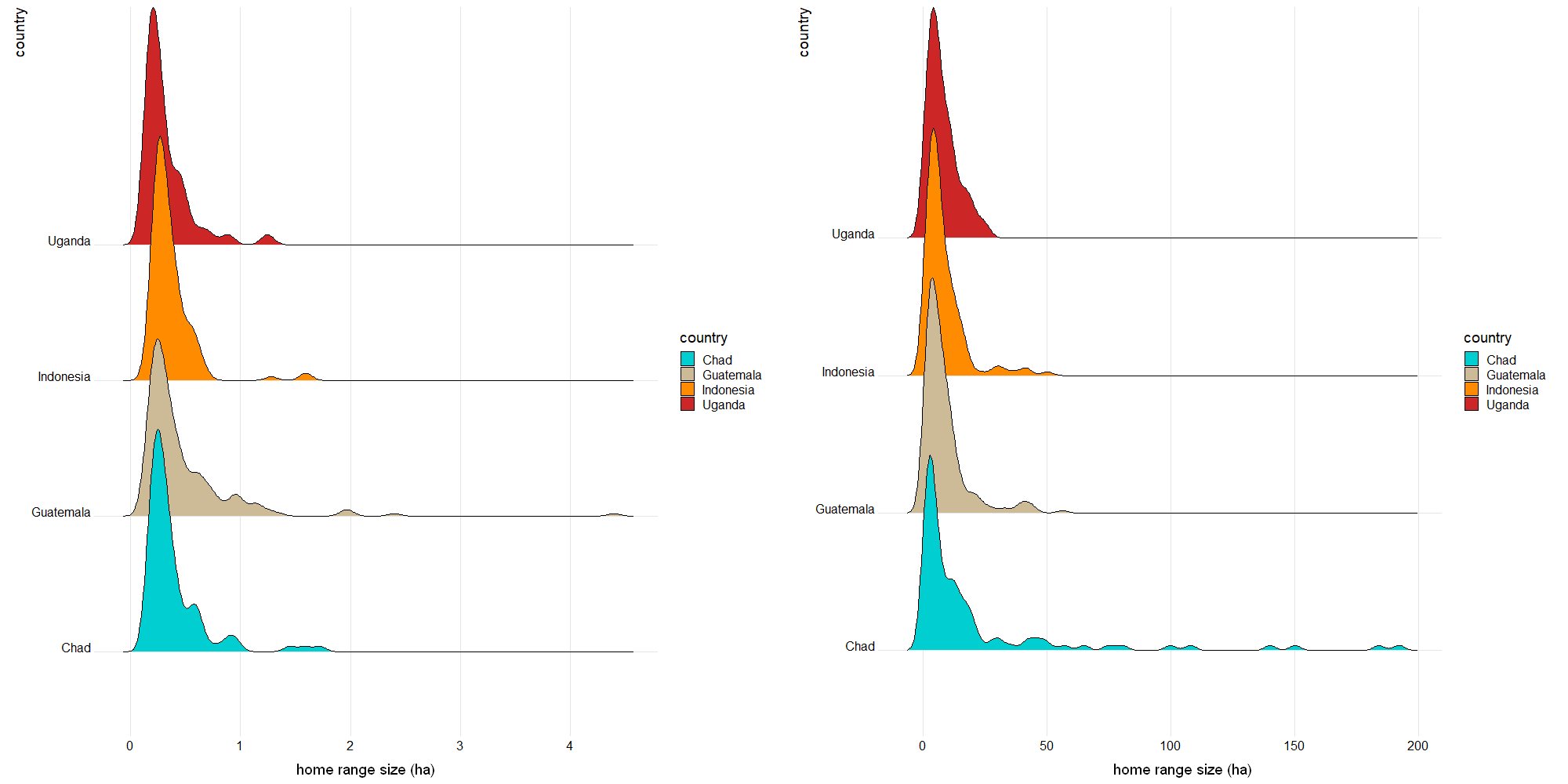
**

**Supplementary Figure 2**. Core (left) and extended (right) home size density distribution of dogs collared in Chad, Guatemala, Indonesia and Uganda between January 2018 and February 2019.

**Supplementary Table 1.** Detailed home range and regression models results. BCS: body condition score, FRT: free-roaming time.

|  |  |  | **Chad** | **Guatemala** | **Indonesia** | **Uganda** |
| --- | --- | --- | --- | --- | --- | --- |
| Number of collars with useable data | | | 100 | 254 | 149 | 95 |
| Number of collars used to calculate home range size^1^ | | | 94 | 200 | 128 | 89 |
| Mean core home range in ha (95% range) | | | 0.30 (0.17 – 0.92) | 0.33 (0.17 -1.1) | 0.30 (0.20 – 0.61) | 0.25 (0.15 -0.72) |
| Mean extended home range in ha (95% range) | | | 7.7 (1.1 -103) | 5.7 (1.5 -27.5) | 5.6 (1.6 – 26.5) | 5.7 (1.3 – 19.1) |
|  | | |  |  |  |  |
| **Factors selected within the best models with the core home range as output^2^** | | | | | | |
| Sex | Male | Baseline | - | - | - | - |
|  | Female | Coefficient | -0.33 | -0.13 | 0.06 | 0.04 |
|  |  | SE | 0.12 | 0.10 | 0.07 | 0.13 |
|  |  | p-value | **0.005** | 0.172 | 0.411 | 0.756 |
| Age | Adult | Baseline |  |  | - | - |
|  | Young | Coefficient | Not investigated | Not investigated | -0.15 | -0.26 |
|  |  | SE |  |  | 0.07 | 0.21 |
|  |  | p-value |  |  | **0.032** | 0.224 |
|  | Senior | Coefficient |  |  | -0.24 | -0.39 |
|  |  | SE |  |  | 0.21 | 0.18 |
|  |  | p-value |  |  | 0.265 | **0.031** |
| BCS | Level 3 | Baseline |  | - |  | - |
|  | Level 1 | Coefficient |  | -0.35 |  |  |
|  |  | SE |  | 0.17 |  | Not investigated |
|  |  | p-value |  | **0.038** |  |  |
|  | Level 2 | Coefficient | Not investigated | -0.14 | Not investigated | -0.28 |
|  |  | SE |  | 0.10 |  | 0.18 |
|  |  | p-value |  | 0.145 |  | 0.132 |
|  | Level 4 | Coefficient |  | 0.24 |  | -0.41 |
|  |  | SE |  | 0.23 |  | 0.23 |
|  |  | p-value |  | 0.314 |  | 0.078 |
| Being a guardian dog | No | Baseline |  | - | - |  |
|  | Yes | Coefficient | Not investigated | -0.24 | -0.15 | Not investigated |
|  |  | SE |  | 0.18 | 0.27 |  |
|  |  | p-value |  | 0.177 | 0.586 |  |
| Being a hunting dog | No | Baseline | - | - |  |  |
|  | Yes | Coefficient | 0.71 | 0.12 | Not investigated | Not investigated |
|  |  | SE | 0.36 | 0.15 |  |  |
|  |  | p-value | 0.051 | 0.415 |  |  |
| Being a shepherd dog | No | Baseline | - |  |  | - |
|  | Yes | Coefficient | -0.06 | Not investigated | Not investigated | 0.66 |
|  |  | SE | 0.11 |  |  | 0.24 |
|  |  | p-value | 0.585 |  |  | **0.007** |
| Raised for meat consumption | No | Baseline |  |  | - |  |
|  | Yes | Coefficient | Not investigated | Not investigated | -0.02 | Not investigated |
|  |  | SE |  |  | 0.09 |  |
|  |  | p-value |  |  | 0.786 |  |
| FRT | Always | Baseline |  |  | - |  |
|  | Day | Coefficient |  |  | 0.19 |  |
|  |  | SE |  |  | 0.16 |  |
|  |  | p-value |  |  | 0.250 |  |
|  | Night | Coefficient | Not selected in the | Not investigated | -0.27 | Not selected in the |
|  |  | SE | "best models" |  | 0.37 | "best models" |
|  |  | p-value |  |  | 0.455 |  |
|  | Sometimes | Coefficient |  |  | -0.42 |  |
|  |  | SE |  |  | 0.12 |  |
|  |  | p-value |  |  | **0.001** |  |
| Study site | Danamadji | Baseline | - |  |  |  |
|  | Yao | Coefficient | -0.16 |  |  |  |
|  |  | SE | 0.12 |  |  |  |
|  |  | p-value | 0.177 |  |  |  |
|  | Poptún | Baseline |  | - |  |  |
|  | Romana | Coefficient |  | 0.32 |  |  |
|  |  | SE |  | 0.13 |  |  |
|  |  | p-value |  | **0.014** |  |  |
|  | Sabaneta | Coefficient |  | 0.20 |  |  |
|  |  | SE |  | 0.11 |  |  |
|  |  | p-value |  | 0.058 |  |  |
|  | Habi | Baseline |  |  |  |  |
|  | Hepang | Coefficient |  |  |  |  |
|  |  | SE |  |  | Not selected in the |  |
|  |  | p-value |  |  | "best models" |  |
|  | Pogon | Coefficient |  |  |  |  |
|  |  | SE |  |  |  |  |
|  |  | p-value |  |  |  |  |
|  | Soroti | Baseline |  |  |  |  |
|  | Kamuda 1 | Coefficient |  |  |  |  |
|  |  | SE |  |  |  | Not selected in the |
|  |  | p-value |  |  |  | "best models" |
|  | Kamuda 2 | Coefficient |  |  |  |  |
|  |  | SE |  |  |  |  |
|  |  | p-value |  |  |  |  |
|  |  |  |  |  |  |  |
| **Factors selected within the best models with the extended home range as output^2^** | | | | | | |
| Sex | Male | Baseline | - | - | - | - |
|  | Female | Coefficient | -0.42 | -0.12 | 0.12 | 0.17 |
|  |  | SE | 0.32 | 0.13 | 0.15 | 0.21 |
|  |  | p-value | 0.186 | 0.366 | 0.406 | 0.423 |
| Age | Adult | Baseline |  |  | - | - |
|  | Young | Coefficient | Not investigated | Not investigated | -0.35 | -0.80 |
|  |  | SE |  |  | 0.15 | 0.34 |
|  |  | p-value |  |  | **0.020** | **0.021** |
|  | Senior | Coefficient |  |  | 0.31 | -0.43 |
|  |  | SE |  |  | 0.46 | 0.28 |
|  |  | p-value |  |  | 0.508 | 0.129 |
| BCS | Level 3 | Baseline |  |  |  | - |
|  | Level 1 | Coefficient |  |  |  |  |
|  |  | SE |  |  |  | Not investigated |
|  |  | p-value |  |  | Not investigated |  |
|  | Level 2 | Coefficient | Not investigated | Not selected in the |  | -0.07 |
|  |  | SE |  | "best models" |  | 0.28 |
|  |  | p-value |  |  |  | 0.810 |
|  | Level 4 | Coefficient |  |  |  | -0.72 |
|  |  | SE |  |  |  | 0.36 |
|  |  | p-value |  |  |  | 0.053 |
| Being a guardian dog | No | Baseline |  | - | - |  |
|  | Yes | Coefficient | Not investigated | -0.23 | -0.48 |  |
|  |  | SE |  | 0.24 | 0.56 |  |
|  |  | p-value |  | 0.345 | 0.396 |  |
| Being a hunting dog | No | Baseline | - | - |  |  |
|  | Yes | Coefficient | 2.04 | 0.23 | Not investigated | Not investigated |
|  |  | SE | 0.97 | 0.20 |  |  |
|  |  | p-value | **0.04** | 0.237 |  |  |
| Being a shepherd dog | No | Baseline | - |  |  | - |
|  | Yes | Coefficient | 0.36 | Not investigated | Not investigated | 0.34 |
|  |  | SE | 0.33 |  |  | 0.38 |
|  |  | p-value | 0.276 |  |  | 0.375 |
| Raised for meat consumption | No | Baseline |  |  | - |  |
|  | Yes | Coefficient | Not investigated | Not investigated | -0.03 | Not investigated |
|  |  | SE |  |  | 0.20 |  |
|  |  | p-value |  |  | 0.867 |  |
| FRT | Always | Baseline |  |  | - |  |
|  | Day | Coefficient |  |  | -0.35 |  |
|  |  | SE |  |  | 0.36 |  |
|  |  | p-value |  |  | 0.332 |  |
|  | Night | Coefficient | Not selected in the | Not investigated | -0.99 | Not selected in the |
|  |  | SE | "best models" |  | 0.77 | "best models" |
|  |  | p-value |  |  | 0.204 |  |
|  | Sometimes | Coefficient |  |  | -0.63 |  |
|  |  | SE |  |  | 0.27 |  |
|  |  | p-value |  |  | **0.021** |  |
| Study site | Danamadji | Baseline | - |  |  |  |
|  | Yao | Coefficient | -0.48 |  |  |  |
|  |  | SE | 0.28 |  |  |  |
|  |  | p-value | 0.095 |  |  |  |
|  | Poptún | Baseline |  | - |  |  |
|  | Romana | Coefficient |  | 0.59 |  |  |
|  |  | SE |  | 0.17 |  |  |
|  |  | p-value |  | **0.001** |  |  |
|  | Sabaneta | Coefficient |  | 0.22 |  |  |
|  |  | SE |  | 0.14 |  |  |
|  |  | p-value |  | 0.114 |  |  |
|  | Habi | Baseline |  |  | - |  |
|  | Hepang | Coefficient |  |  | -0.29 |  |
|  |  | SE |  |  | 0.17 |  |
|  |  | p-value |  |  | 0.101 |  |
|  | Pogon | Coefficient |  |  | 0.48 |  |
|  |  | SE |  |  | 0.20 |  |
|  |  | p-value |  |  | **0.020** |  |
|  | Soroti | Baseline |  |  |  |  |
|  | Kamuda 1 | Coefficient |  |  |  |  |
|  |  | SE |  |  |  | Not selected in the |
|  |  | p-value |  |  |  | "best models" |
|  | Kamuda 2 | Coefficient |  |  |  |  |
|  |  | SE |  |  |  |  |
|  |  | p-value |  |  |  |  |

^1^Dogs whose collar recorded less than 50 GPS fixes or for less than 24 hours were excluded from HR analysis.

^2^If a factor was selected in several best models, the coefficient, SE and p-value was extracted from the model with the lowest AIC

**Supplementary Table 2.** List of factors included the "best models" selected in each country. Models are ranked by AIC. All other models have a ΔAIC lower than two. BCS: body condition score, FRT: free-roaming time.

| **Model output** | **Rank** | **Chad** | **Guatemala** | **Indonesia** | **Uganda** |
| --- | --- | --- | --- | --- | --- |
| Logarithm of the core home range | 1 | sex + being a hunting dog | BCS + study site | age + FRT | age + BCS + being a shepherd dog |
|  | 2 | sex + being a hunting dog + study site | sex + BCS + study site | sex + age + FRT | age + being a shepherd dog |
|  | 3 | sex + study site | BCS + being a guardian dog + study site | FRT | being a shepherd dog |
|  | 4 | sex + being a shepherd dog + being a hunting dog | sex + BCS + being a guardian dog + study site | age + being a guardian dog + FRT | sex + age + BCS + being a shepherd dog |
|  | 5 | being a hunting dog + study site | study site | age + raised for meat + FRT |  |
|  | 6 | sex | BCS + being a hunting dog + study site |  |  |
|  | 7 |  | sex + BCS + being a hunting dog + study site |  |  |
|  | 8 |  | BCS + being a hunting dog + being a guardian dog + study site |  |  |
|  | 9 |  | BCS + being a hunting dog |  |  |
|  | 10 |  | being a guardian dog + study site |  |  |
|  |  |  |  |  |  |
| Logarithm of the extended home range | 1 | being a hunting dog + study site | study site | age + FRT + study site | age + BCS |
|  | 2 | being a shepherd dog + being a hunting dog + study site | being a hunting dog + study site | age + being a guardian dog + FRT+ study site | age |
|  | 3 | being a hunting dog | being a guardian dog + study site | sex + age + FRT + study site | age + BCS + being a shepherd dog |
|  | 4 | sex + being a hunting dog | sex + study site | age + study site | age + being shepherd dog |
|  | 5 | sex + being a hunting dog + study site | being a hunting dog + being a guardian dog + study site | age + being raised for meat + FRT + study site | sex + age |
|  | 6 |  | sex + being a hunting dog + study site |  | sex + age + BCS |

**Table S3.** Detailed regression models results restricted to dogs whose collar recorded for at least 48 hours. BCS: body condition score, FRT: free-roaming time.

|  |  |  | **Chad** | **Guatemala** | **Indonesia** | **Uganda** |
| --- | --- | --- | --- | --- | --- | --- |
| **Factors selected within the best models with the core home range as output^1^** | | | | | | |
| Sex | Male | Baseline | - | - | - | - |
|  | Female | Coefficient | -0.31 | -0.11 | 0.04 | 0.15 |
|  |  | SE | 0.12 | 0.10 | 0.08 | 0.13 |
|  |  | p-value | **0.010** | 0.263 | 0.615 | 0.245 |
| Age | Adult | Baseline |  |  | - | - |
|  | Young | Coefficient | Not investigated | Not investigated | -0.15 | -0.22 |
|  |  | SE |  |  | 0.07 | 0.20 |
|  |  | p-value |  |  | **0.033** | 0.279 |
|  | Senior | Coefficient |  |  | -0.23 | -0.33 |
|  |  | SE |  |  | 0.21 | 0.17 |
|  |  | p-value |  |  | 0.279 | 0.056 |
| BCS | Level 3 | Baseline |  | - |  | - |
|  | Level 1 | Coefficient |  | -0.27 |  |  |
|  |  | SE |  | 0.17 |  | Not investigated |
|  |  | p-value |  | 0.107 |  |  |
|  | Level 2 | Coefficient | Not investigated | -0.15 | Not investigated | -0.22 |
|  |  | SE |  | 0.10 |  | 0.18 |
|  |  | p-value |  | 0.119 |  | 0.212 |
|  | Level 4 | Coefficient |  | 0.22 |  | -0.50 |
|  |  | SE |  | 0.24 |  | 0.23 |
|  |  | p-value |  | 0.358 |  | **0.033** |
| Being a guardian dog | No | Baseline |  | - | - |  |
|  | Yes | Coefficient | Not investigated | -0.13 | -0.16 | Not investigated |
|  |  | SE |  | 0.18 | 0.26 |  |
|  |  | p-value |  | 0.482 | 0.556 |  |
| Being a hunting dog | No | Baseline | - | - |  |  |
|  | Yes | Coefficient | 0.73 | 0.03 | Not investigated | Not investigated |
|  |  | SE | 0.36 | 0.14 |  |  |
|  |  | p-value | **0.045** | 0.834 |  |  |
| Being a shepherd dog | No | Baseline | - |  |  | - |
|  | Yes | Coefficient | -0.04 | Not investigated | Not investigated | 0.62 |
|  |  | SE | 0.11 |  |  | 0.25 |
|  |  | p-value | 0.729 |  |  | **0.015** |
| Raised for meat consumption | No | Baseline |  |  | - |  |
|  | Yes | Coefficient | Not investigated | Not investigated | -0.06 | Not investigated |
|  |  | SE |  |  | 0.09 |  |
|  |  | p-value |  |  | 0.509 |  |
| FRT | Always | Baseline |  |  | - | - |
|  | Day | Coefficient |  |  | 0.07 | -0.51 |
|  |  | SE |  |  | 0.18 | 0.50 |
|  |  | p-value |  |  | 0.691 | 0.311 |
|  | Night | Coefficient | Not selected in the | Not investigated | -0.26 | 0.20 |
|  |  | SE | "best models" |  | 0.36 | 0.14 |
|  |  | p-value |  |  | 0.477 | 0.164 |
|  | Sometimes | Coefficient |  |  | -0.41 | -0.27 |
|  |  | SE |  |  | 0.12 | 0.24 |
|  |  | p-value |  |  | **0.001** | 0.251 |
| Study site | Danamadji | Baseline | - |  |  |  |
|  | Yao | Coefficient | -0.15 |  |  |  |
|  |  | SE | 0.12 |  |  |  |
|  |  | p-value | 0.228 |  |  |  |
|  | Poptún | Baseline |  | - |  |  |
|  | Romana | Coefficient |  | 0.33 |  |  |
|  |  | SE |  | 0.13 |  |  |
|  |  | p-value |  | **0.010** |  |  |
|  | Sabaneta | Coefficient |  | 0.10 |  |  |
|  |  | SE |  | 0.10 |  |  |
|  |  | p-value |  | 0.331 |  |  |
|  | Habi | Baseline |  |  | - |  |
|  | Hepang | Coefficient |  |  | -0.05 |  |
|  |  | SE |  |  | 0.08 |  |
|  |  | p-value |  |  | 0.604 |  |
|  | Pogon | Coefficient |  |  | 0.11 |  |
|  |  | SE |  |  | 0.11 |  |
|  |  | p-value |  |  | 0.298 |  |
|  | Soroti | Baseline |  |  |  |  |
|  | Kamuda 1 | Coefficient |  |  |  |  |
|  |  | SE |  |  |  | Not selected in the |
|  |  | p-value |  |  |  | "best models" |
|  | Kamuda 2 | Coefficient |  |  |  |  |
|  |  | SE |  |  |  |  |
|  |  | p-value |  |  |  |  |
|  |  |  |  |  |  |  |
| **Factors selected within the best models with the extended home range as output^1^** | | | | | | |
| Sex | Male | Baseline | - | - | - | - |
|  | Female | Coefficient | -0.46 | -0.15 | 0.02 | 0.26 |
|  |  | SE | 0.33 | 0.13 | 0.16 | 0.22 |
|  |  | p-value | 0.164 | 0.267 | 0.886 | 0.244 |
| Age | Adult | Baseline |  |  | - | - |
|  | Young | Coefficient | Not investigated | Not investigated | -0.29 | -0.76 |
|  |  | SE |  |  | 0.15 | 0.34 |
|  |  | p-value |  |  | 0.053 | **0.029** |
|  | Senior | Coefficient |  |  | 0.26 | -0.38 |
|  |  | SE |  |  | 0.44 | 0.28 |
|  |  | p-value |  |  | 0.560 | 0.186 |
| BCS | Level 3 | Baseline |  |  |  | - |
|  | Level 1 | Coefficient |  |  |  |  |
|  |  | SE |  |  |  | Not investigated |
|  |  | p-value |  |  |  |  |
|  | Level 2 | Coefficient | Not investigated | Not selected in the | Not investigated | -0.02 |
|  |  | SE |  | "best models" |  | 0.28 |
|  |  | p-value |  |  |  | 0.941 |
|  | Level 4 | Coefficient |  |  |  | -0.86 |
|  |  | SE |  |  |  | 0.39 |
|  |  | p-value |  |  |  | **0.032** |
| Being a guardian dog | No | Baseline |  | - | - |  |
|  | Yes | Coefficient | Not investigated | -0.16 | -0.50 | Not investigated |
|  |  | SE |  | 0.26 | 0.53 |  |
|  |  | p-value |  | 0.527 | 0.352 |  |
| Being a hunting dog | No | Baseline | - | - |  |  |
|  | Yes | Coefficient | 2.05 | 0.15 | Not investigated | Not investigated |
|  |  | SE | 0.99 | 0.20 |  |  |
|  |  | p-value | **0.041** | 0.453 |  |  |
| Being a shepherd dog | No | Baseline | - |  |  | - |
|  | Yes | Coefficient | 0.39 | Not investigated | Not investigated | 0.32 |
|  |  | SE | 0.33 |  |  | 0.42 |
|  |  | p-value | 0.249 |  |  | 0.452 |
| Raised for meat consumption | No | Baseline |  |  | - |  |
|  | Yes | Coefficient | Not investigated | Not investigated | -0.03 | Not investigated |
|  |  | SE |  |  | 0.20 |  |
|  |  | p-value |  |  | 0.870 |  |
| FRT | Always | Baseline |  |  | - |  |
|  | Day | Coefficient |  |  | -0.35 |  |
|  |  | SE |  |  | 0.39 |  |
|  |  | p-value |  |  | 0.373 |  |
|  | Night | Coefficient | Not selected in the | Not investigated | -0.93 | Not selected in the |
|  |  | SE | "best models" |  | 0.74 | "best models" |
|  |  | p-value |  |  | 0.209 |  |
|  | Sometimes | Coefficient |  |  | -0.55 |  |
|  |  | SE |  |  | 0.26 |  |
|  |  | p-value |  |  | **0.036** |  |
| Study site | Danamadji | Baseline | - |  |  |  |
|  | Yao | Coefficient | -0.49 |  |  |  |
|  |  | SE | 0.29 |  |  |  |
|  |  | p-value | 0.101 |  |  |  |
|  | Poptún | Baseline |  | - |  |  |
|  | Romana | Coefficient |  | 0.60 |  |  |
|  |  | SE |  | 0.18 |  |  |
|  |  | p-value |  | **0.001** |  |  |
|  | Sabaneta | Coefficient |  | 0.23 |  |  |
|  |  | SE |  | 0.15 |  |  |
|  |  | p-value |  | 0.111 |  |  |
|  | Habi | Baseline |  |  | - |  |
|  | Hepang | Coefficient |  |  | -0.19 |  |
|  |  | SE |  |  | 0.17 |  |
|  |  | p-value |  |  | 0.281 |  |
|  | Pogon | Coefficient |  |  | 0.67 |  |
|  |  | SE |  |  | 0.22 |  |
|  |  | p-value |  |  | **0.003** |  |
|  | Soroti | Baseline |  |  |  |  |
|  | Kamuda 1 | Coefficient |  |  |  |  |
|  |  | SE |  |  |  | Not selected in the |
|  |  | p-value |  |  |  | "best models" |
|  | Kamuda 2 | Coefficient |  |  |  |  |
|  |  | SE |  |  |  |  |
|  |  | p-value |  |  |  |  |

^1^If a factor was selected in several best models, the coefficient, SE and p-value was extracted from the model with the lowest AIC
